# Improving Maternal Care through a State-Wide Health Insurance Program: A Cost and Cost-Effectiveness Study in Rural Nigeria

**DOI:** 10.1371/journal.pone.0139048

**Published:** 2015-09-28

**Authors:** Gabriela B. Gomez, Nicola Foster, Daniella Brals, Heleen E. Nelissen, Oladimeji A. Bolarinwa, Marleen E. Hendriks, Alexander C. Boers, Diederik van Eck, Nicole Rosendaal, Peju Adenusi, Kayode Agbede, Tanimola M. Akande, Michael Boele van Hensbroek, Ferdinand W. Wit, Catherine A. Hankins, Constance Schultsz

**Affiliations:** 1 Department of Global Health/Amsterdam Institute for Global Health and Development, Academic Medical Center, University of Amsterdam, Amsterdam, The Netherlands; 2 Department of Global Health and Development, London School of Hygiene and Tropical Medicine, London, United Kingdom; 3 Health Economics Unit, School of Public Health and Family Medicine, University of Cape Town, Cape Town, South Africa; 4 Department of Epidemiology and Community Health, University of Ilorin Teaching Hospital, Ilorin, Nigeria; 5 Pharmaccess Foundation, Amsterdam, The Netherlands; 6 Hygeia Nigeria Ltd, Lagos, Nigeria; 7 Ogo Oluwa Hospital, Bacita, Kwara State, Nigeria; 8 Global Child Health Group, Emma Children's Hospital AMC, Amsterdam, The Netherlands; 9 Department of Infectious Disease Epidemiology, London School of Hygiene and Tropical Medicine, London, United Kingdom; Erasmus University Rotterdam, NETHERLANDS

## Abstract

**Background:**

While the Nigerian government has made progress towards the Millennium Development Goals, further investments are needed to achieve the targets of post-2015 Sustainable Development Goals, including Universal Health Coverage. Economic evaluations of innovative interventions can help inform investment decisions in resource-constrained settings. We aim to assess the cost and cost-effectiveness of maternal care provided within the new Kwara State Health Insurance program (KSHI) in rural Nigeria.

**Methods and Findings:**

We used a decision analytic model to simulate a cohort of pregnant women. The primary outcome is the incremental cost effectiveness ratio (ICER) of the KSHI scenario compared to the current standard of care. Intervention cost from a healthcare provider perspective included service delivery costs and above-service level costs; these were evaluated in a participating hospital and using financial records from the managing organisations, respectively. Standard of care costs from a provider perspective were derived from the literature using an ingredient approach. We generated 95% credibility intervals around the primary outcome through probabilistic sensitivity analysis (PSA) based on a Monte Carlo simulation. We conducted one-way sensitivity analyses across key model parameters and assessed the sensitivity of our results to the performance of the base case separately through a scenario analysis. Finally, we assessed the sustainability and feasibility of this program’s scale up within the State’s healthcare financing structure through a budget impact analysis. The KSHI scenario results in a health benefit to patients at a higher cost compared to the base case. The mean ICER (US$46.4/disability-adjusted life year averted) is considered very cost-effective compared to a willingness-to-pay threshold of one gross domestic product per capita (Nigeria, US$ 2012, 2,730). Our conclusion was robust to uncertainty in parameters estimates (PSA: median US$49.1, 95% credible interval 21.9–152.3), during one-way sensitivity analyses, and when cost, quality, cost and utilization parameters of the base case scenario were changed. The sustainability of this program’s scale up by the State is dependent on further investments in healthcare.

**Conclusions:**

This study provides evidence that the investment made by the KSHI program in rural Nigeria is likely to have been cost-effective; however, further healthcare investments are needed for this program to be successfully expanded within Kwara State. Policy makers should consider supporting financial initiatives to reduce maternal mortality tackling both supply and demand issues in the access to care.

## Introduction

In 2000, governments stated their commitment to Millennium Development Goal (MDG) 5 to reduce the maternal mortality ratio by three quarters between 1990 and 2015 [1]. More recently, governments reaffirmed their commitment to reducing maternal mortality with a proposed new target of fewer than 70 maternal deaths per 100,000 live births by 2030 and expanded the scope of the post-2015 Sustainable Development Goal (SDG) 3 to include achieving Universal Health Coverage [2]. However, attaining such targets will be challenging due to barriers in service utilisation and access to good quality care in many settings. In 2010, it was estimated that Nigeria alone accounted for 14% of maternal deaths worldwide [3]. With a national estimate of 224 deaths per 100,000 live births in 2013, a Nigerian woman has, currently, a lifetime risk for maternal death of one in 79 [4]. While the Nigerian government is making progress in improving maternal health, further investments might be needed to achieve MDG5 and to make progress towards SDG3.

Several key health interventions aim to improve both the quality of available healthcare services and the demand for those services [5–15]. However, the uptake of evidence-based interventions to reduce maternal mortality is limited by user acceptability and affordability factors [16–18], whereas their implementation is limited by availability of resources and health system constraints [19].

The objectives of the Health Insurance Fund [20], funded by the Dutch Ministry of Foreign Affairs, are to “1) increase access to quality basic healthcare for currently uninsured groups, mainly through private health facilities; 2) evaluate different private healthcare delivery models based on a demand-driven and results-oriented approach; 3) directly support Millennium Development Goals (MDG) 1 and 6: reducing poverty and halting the spread of HIV/AIDS, tuberculosis, malaria and other major diseases; 4) lower the threshold for investment in private healthcare infrastructure; and 5) build sustainable medical and financial-administrative capacity in the health sector” [20]. It is within this framework that the Kwara State Health Insurance (KSHI) was created in 2007 as a public-private partnership between the Kwara State Government, Hygeia Community Health Care, the Health Insurance Fund, and PharmAccess. The objective is to improve access to affordable and quality care for low-income people in Kwara State, prioritizing those earning less than US$1.5 per day. The program tackles both demand and supply aspects of the healthcare system simultaneously by subsidizing insurance coverage and improving the quality of care in the participating healthcare facilities through structural upgrading, training staff in guideline-based care, and supporting hospital management.

By January 2015, 85,110 people had enrolled in the KSHI program. Impact results two years after implementation showed an increase in utilisation of healthcare and a decrease in all out-of-pocket (OOP) healthcare expenditures for those living in areas where the insurance is offered [21]. The KSHI program has also been associated with a significant decrease in blood pressure in hypertensive patients living in areas where the insurance is offered [22].

In this study, we aim to assess whether the implementation of the KSHI program, including the initial investment by donors to establish this program, is likely to have been a cost-effective maternal care intervention in rural Nigeria. We use empirically-collected information from impact and costing studies undertaken during implementation of the program, as well as insurance and hospital monitoring databases.

## Methods

We used a decision analytic model to simulate a cohort of pregnant women, followed down a pathway of care during their current pregnancy until delivery. We defined two scenarios in our primary analysis: 1) current standard of care (base case scenario) where women do not have access to benefits from the insurance program; and 2) KSHI scenario (intervention scenario) where women have access to the insurance and to hospitals participating in the KSHI program. Alternative base case scenarios were defined in a scenario analysis, in addition to the primary analysis above, comparing: 3) an increased utilization of the standard of care clinics; 4) an increased cost and quality of care improvement in the standard of care clinics; and 5) increased utilization, increased cost and quality of care improvement in the standard of care clinics. The model's primary outcome is the incremental cost per disability adjusted life year (DALY) averted in the KSHI scenario compared to the base case scenario. This incremental cost-effectiveness ratio (ICER) was calculated as the ratio of the difference in costs and DALYs averted between the intervention and base case scenarios. The ICER was then compared to a country-specific willingness-to-pay (WTP) threshold, defined as a country's per capita gross domestic product (GDP) [23]. For Nigeria, the GDP per capita was US$ 2,730 in 2012 [24]. If the ICER is below this WTP threshold, the intervention is considered very cost-effective. Key model input parameters are shown in Table 1 and further details can be found in the S1 File.

**Table 1:** Input parameters for cost-effectiveness analyses.

	Standard of care	distr	Ref	KSHI care	distr	ref
**Utilization**						
Access to ANC	0.6–0.7	uniform	[57,58]	0.8–0.9	uniform	[58]
Delivery in health facility (all)	0.4–0.6	uniform	[4,57,58]	0.65–0.7	uniform	[58]
Access to EOC, if delivery in health facility	0.9–0.95	uniform	[59]	1		
Delivery in health facility, if previous ANC	0.96 (0.03)	beta	[57]			
Delivery at home, if previous ANC	0.05 (0.03)	beta	[57]			
Access to EOC, if delivery at home	0.136 (0.02)	beta	[27]			
**Outcomes**						
Haemorrhage	0.051 (0.04)	beta	[60]			
Anaemia among those surviving an haemorrhagic episode	0.12 (0.01)	beta	[61]			
Death following haemorrhage	0.028–0.273	uniform	[62]			
rr haemorrhage, if EOC	0.34 (0.19)	beta	[63]			
rr anaemia, if EOC	0.5 (0.14)	beta	[63]			
Sepsis	0.017 (0.01–0.03)	triangular	[31]			
Secondary infertility among those surviving following sepsis	0.05–0.1	uniform	[61]			
Death following sepsis	0–0.727	uniform	[62]			
rr sepsis if delivery at hospital	0.54 (0.4–0.65)	triangular	[33]			
Obstructed labour (OL)	0.06 (0.02)	beta	[34,64]			
Fistula among those surviving, if no EOC following OL	0.14 (0.01)	beta	[34]			
Death, following OL, if no EOC	0.007 (0.01)	beta	[61]			
Hypertensive disorders (HTD)	0.085 (0.04)	beta	[65]			
Death, following a HTD	0.083 (0.02)	beta	[65]			
rr HTD if ANC	—	—	—	0.41 (0.08)	beta	[66]
**Cost** (US$2012)									
Cost, ANC	12.4–61.5	uniform	*			
Cost, delivery no complications	9.65–27.2	uniform	*			
Cost, delivery complications	46.7–53.3	uniform	*			
Cost, treatment of fistula	190.9–382.7	uniform	[67,68]			
Cost, treatment of anaemia	9.81–13.79	uniform	*			
**DALY**									
DALYs, death	23.43 (21.09–25.77)	triangular	*			
DALYs, anaemia	0.09 (0.08–0.09)	triangular	*			
DALYs, infertility	0.1 (0.09–0.11)	triangular	*			
DALYs, fistula	10.93 (9.84–12.02)	triangular	*			

ANC, antenatal care; EOC, essential obstetric care; distr: probability distribution specified for each parameter in the Monte Carlo simulations; ref, reference; rr, relative risk; OL, obstructed labour; HTD, hypertensive disorder. Beta distributions are specified by mean (standard deviation); uniform distributions by minimum and maximum values; triangular distributions by average (minimum and maximum).

*Own calculation (S1 File).

### Base case (standard of care)

The current standard of care in rural Nigeria (base case scenario) was characterized in two dimensions: utilization and quality of care. Kwara State has a health system with inadequate government funding, weak governance and legislation, and poor health infrastructure and service quality. The State is participating in the federally-funded National Health Insurance Scheme (NHIS). The majority of enrollees are individuals working in the formal sector. The NHIS started a rural community-based social health insurance program in 2010 but access to this scheme is limited [21]. Data collected during the baseline survey of the KSHI impact evaluation in 2009 showed that less than 1% of the population in the area was enrolled in any health insurance scheme [21]. The base case is therefore defined as a regionally-representative situation where functional health care facilities are mainly primary care clinics with limited access to secondary care (such as surgery, inpatient care). The assumptions on utilization and quality of care derived from regionally representative surveys, maternal health audits, and data collection as part of the baseline survey of the KSHI impact evaluation in 2009 [21]. All assumptions are described in the S1 File; key parameters and sources are presented in Table 1.

### Intervention (KSHI program)

The intervention modelled is the KSHI program. This includes a subsidized health insurance covering access to comprehensive health care, including primary care; treatment for malaria, tuberculosis, and HIV opportunistic infections; maternal and child care; surgeries; and care for chronic diseases. It also includes upgrades to facilities and technical assistance in program management by PharmAccess Foundation. In this context, the impact of the KSHI program is hypothesised to result from two pathways: 1) increased utilization of maternal services, defined as antenatal care (ANC) visits, delivery in health facilities, and emergency obstetric care (EOC) when complications during delivery arise; and 2) increased quality of care of maternal services provided (access to more facilities offering EOC and preventive treatment of hypertensive disorders complications during ANC) [11,25].

### Model description

The model explicitly considers utilization and composition of ANC, the location of the care accessed, and the type of assistance provided during the delivery as well as availability of EOC. Essential obstetric care is defined as care including capacity to administrate parenteral antibiotics, parenteral oxytocic drugs, and parenteral anticonvulsants for pre-eclampsia and eclampsia; ability to perform manual removal of placenta and of retained products; ability to perform assisted vaginal delivery, surgery (C-section), and blood transfusions [26]. The schematic representation of the model structure is given in the S1 File.

We considered five clinical outcomes of delivery: post-partum haemorrhage, obstructed labour, hypertensive disorder, sepsis, and uncomplicated delivery. The first four are responsible for the highest proportion of maternal mortality and morbidity in Nigeria [27–29]. We estimated a women’s probability of accessing treatment for these complications to be dependent on the location of care accessed during delivery and whether previous ANC visits were attended during the current pregnancy. Prevalence of adverse delivery outcomes were sourced from systematic reviews or cohort studies specific to Nigeria; when these were not available, we sourced estimates that were regionally representative. With regards to treatment outcome probabilities, all estimates were sourced from clinical trials or meta-analyses of clinical trials [30–34]. Mortality and morbidity outcomes were then translated into years of life lost due to premature mortality (YLLs) and years lived with disability (YLDs), respectively, to calculate total number of DALYs averted using standard methods [35], without age weighting [32]. We measured all costs and DALYs through a time horizon spanning the remaining life expectancy of the cohort (for a detailed description of assumptions, see S1 File).

### Costs

Costs were evaluated from a healthcare provider perspective. For the intervention scenario, we collected data at the Ogo Oluwa Hospital (OOH) in Kwara State. This is a private hospital participating in the KSHI program and serving the community of Bacita, part of Edu local government area (population estimated: 201,642 in 2006 [36]) in the North Central region of Nigeria [37]. The hospital provides ANC and perinatal care as well as EOC. The number of patients enrolled in the KSHI program registered in OOH was 9,738 for the period 2010–2011. These patients represented over 95% of the total number of patients accessing care in OOH (personal communication, medical director OOH).

We measured service delivery costs including costs for building, overhead, staff, equipment and consumables, and maintenance at OOH. The resource use associated with each activity was estimated through observations of practice, a review of financial reporting, and interviews with staff. Resource use measurement took into account the allocation of fixed resources between maternal care and other services. Estimates of drugs and test prices were obtained from suppliers [38]. We extracted information on the total number of pregnancies, ANC visits, and deliveries from insurance claim data for the period covered by the costing exercise. We then calculated costs per ANC consultation and delivery care separately. Finally, we combined the utilisation data with unit costs to calculate the total costs of maternal services at OOH. A sensitivity analysis of assumptions where measurements of parameters were uncertain (percentage mark-up allocated to overheads, staff time, and medical equipment) was undertaken to estimate the impact of these assumptions on our cost estimates.

We also included above-service program costs associated with the local operations of the insurer (Hygeia Nigeria Ltd) and program management at PharmAccess level. The operations at insurer level consist of administration of the package and marketing activities for scaling up of the project. Program management expenses at PharmAccess level consist of expenses related to upgrading of healthcare facilities and technical assistance concerning the health plan. In determining the cost-effectiveness of the program, these costs were taken into account from the beginning of the program in 2006 until 2018. After this date the program is expected to be transferred to the Kwara state Government. Expenses over the period 2006–2013 are audited, while from 2014 the amounts are based on projections. We added this as a mark-up to all patients in the intervention scenario, as this cohort was assumed to be insured. Detailed calculations are given in S1 File.

Finally, we reviewed previous costing studies in Nigeria to validate our cost estimates and provide costs for treatment of morbidities associated with complicated deliveries. When estimates were missing, we used WHO guidelines and unit costs for outpatient visits sourced from WHO-CHOICE [39]. All prices were collected in local currency and are presented in 2012 US$ [40]. Cost information from previous studies was adjusted to account for inflation following standard methods [41,42]. All future costs and outcomes were discounted at 3% per year.

### Budget impact analysis

To explore the sustainability of scaling up the intervention within the current health expenditures in Kwara state, we compared the cost of scaling up the program state-wide, over a five-year period, against current health expenditures for Kwara State, in a separate analysis [43,44].

The size of the population in need of maternal care (women aged 15 to 40 years) was estimated using available demographic data [36] and assuming a population growth equivalent to the rate of natural increase sourced from the World Bank [45]. The same cost assumptions were made for this analysis as in the primary analysis (Table 1). The level of insurance coverage was defined in terms of the proportion of the population in need that access the program. The annual cost of implementing the intervention was based on the estimated number of women in need accessing the program per year. The annual cost of scaling up the maternal care intervention to those in need was calculated using the following equation, excluding any financial gain from cost-sharing revenue collection:

*Population in need of ANC and EOC x unit cost of EOC and ANC (including above service level costs) given prevalence of different complications x insurance coverage—current estimated expenditure on ANC and EOC*

Only the resources and expenditures required above current spending levels were included. Given that the aim of the budget impact analysis is to explore the impact on the State’s health expenditure, the cost to households was not included in this analysis. Unit costs were inflated to 2012 prices when necessary, using the average inflation rate between 1996 and 2014 for Nigeria of 12.33% per annum [46].

### Uncertainty, scenario and sensitivity analyses

Primary results are presented using a probabilistic sensitivity analysis (Monte Carlo simulation) to randomly sample parameters from their probability distributions repeatedly (10,000 times) to generate 95% credibility intervals around the incremental cost per DALY averted [47].

We assessed the sensitivity of our results to the performance of the base case in three ways (further information in S1 File):

1. by varying the utilization of maternal services: this scenario explores the situation where the base case population increases its utilization of standard of care clinics;
2. by varying the costs from the healthcare provider perspective of base case services to those previously reported in the literature [48] and setting the quality of care indicators of the standard of care clinics to high bounds: this scenario explores the situation where the standard of care is financed mainly through the public health system and therefore an increase in costs from a provider perspective is observed, implying an increase in the quality of care due to this increase in investment;
3. by varying the costs from the healthcare provider perspective of base case services to those previously reported in the literature [48], increasing the utilization of services to the standard of care clinics, and setting the quality of care indicators of the standard of care clinics to high bounds: this scenario represents a situation where the increase in funding through the public health system leads to an increase both in quality and in the service utilization in the base case.

Finally, we conducted one-way sensitivity analyses across key model parameters to assess the robustness of our results, varying one parameter at a time between the outer limits of its confidence interval. In particular, we examined the sensitivity of our results to the probability of complications during delivery (by type of complication) as well as to the probabilities of mortality and morbidity from that complication. Similarly, we examined treatment costs for fistula and anaemia, duration of disability for all disabilities, estimates of ANC utilization and delivery at health facilities, and a large variation in the estimates of above-service program costs.

The model was programmed using TreeAge Pro 2014 (TreeAge Software Inc., Williamstown MA), cost analyses were conducted using Microsoft Excel 2013 (Microsoft Corp., Redmond WA). We conducted and present this study following good reporting practices from published standards for reporting of economic evaluations of health interventions, the CHEERS statement, and the Bill and Melinda Gates Foundation, Methods for Economic Evaluation Project [49,50].

### Ethics statement

Empirical costing activities were conducted as part of ongoing evaluation efforts of the Health Insurance Fund program. The main project, QUality Improvement of Cardiovascular care in Kwara (QUICK), was approved on the 30^th^ March 2010 by the ethical review committee at the University of Ilorin Teaching Hospital (reference number: UITH/CAT/189/13/13). We sought an extension of this ethics approval to include ANC and delivery care services data. This extension was granted on the 16^th^ August 2012 by the same ethical review committee (ethical review committee at the University of Ilorin Teaching Hospital, reference number: UITH/CAT/189/15/450). No patient records/information were consulted and patients were not approached during this study. All data were aggregated and anonymized prior to analysis.

## Results

### Service utilisation and maternal care-related costs in OOH

Over 24,000 consultations were recorded during the study period, of which 5,470 (23%) were related to maternal care. In particular, we observed 3.1 to 5 ANC visits per pregnancy and 118 complicated deliveries (19% of total childbirths) during the study period.

**Fig 1:**
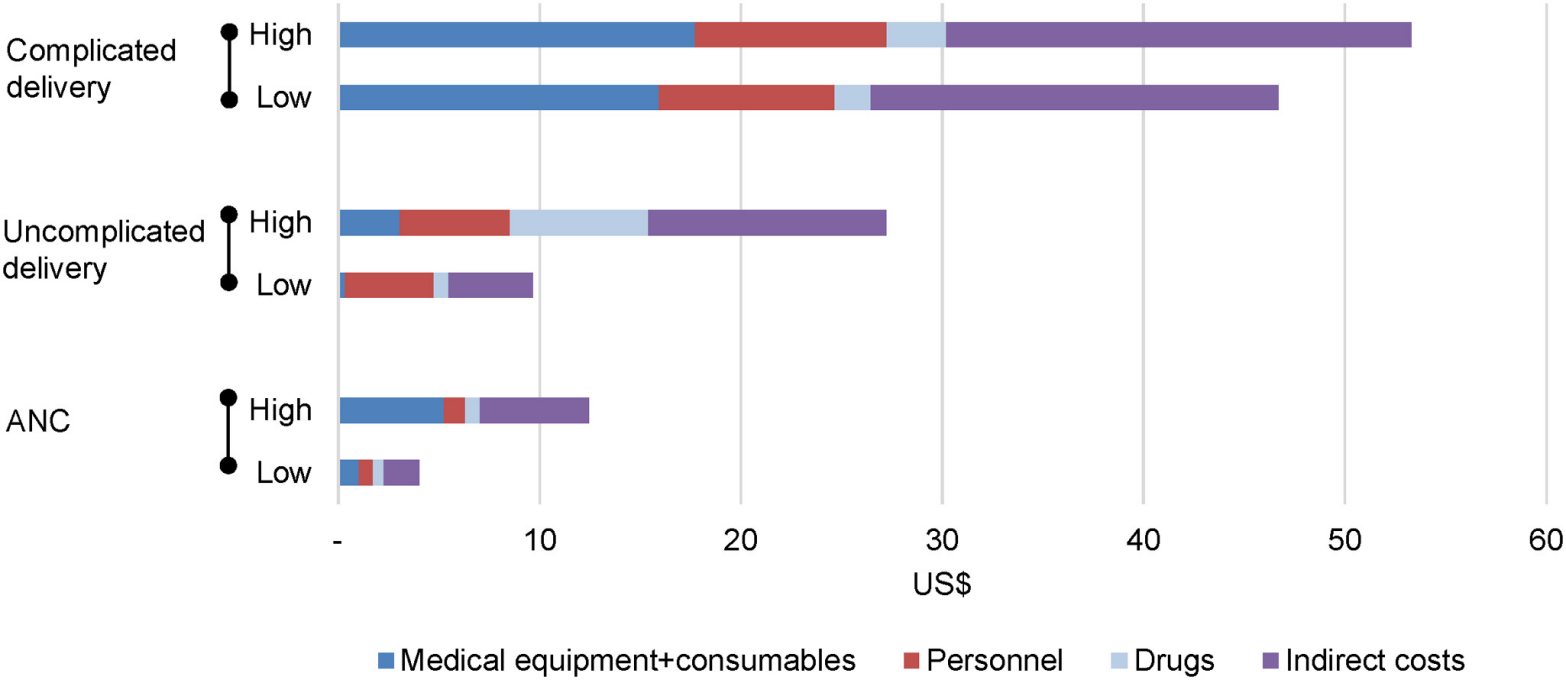
Unit costs by cost category for low and high utilisation profiles. ANC, antenatal care.

The highest unit cost was estimated for complicated deliveries (US$46.7–53.3), followed by uncomplicated deliveries (US$9.65–27.2), and a single ANC visit (US$4.0–12.5). The total ANC cost during pregnancy was estimated at US$12.4–61.5. In Fig 1, we present the unit costs by cost input. For complicated deliveries, the unit cost was driven by the direct costs of equipment, consumables, and personnel; whereas for uncomplicated deliveries, personnel and drug costs largely defined the total unit cost. The estimates for ANC and uncomplicated delivery unit costs were robust to changes in assumptions in the cost sensitivity analysis (S1 File). However, complicated delivery costs were sensitive to variations in the overhead mark-up percentage estimate.

The estimated total cost of ANC services in OOH was US$19,408–60,650; uncomplicated deliveries accounted for a total cost of US$4,825–13,600 and complicated deliveries from US$5,510–6,289. Total cost for maternal services varied from US$29,744 to US$80,539, with ANC services accounting for 65 to 75% of these costs, due to the high level of utilisation. The average annualised above-service program cost calculated over the full period 2006–2018 was estimated to be US$ 24.1 per enrolee.

### Cost-effectiveness of KSHI maternal care

In Table 2, we present the cohort and outcomes distribution for 10,000 pregnant women simulated for each scenario.

**Table 2:** Cohort distribution and outcomes.

					PPH	sepsis	HTD	OL	deaths*
		n		n (%)	n	n	n	n	n (%)
**SoC**	no ANC	3,500	complications	751 (21.5)	179	65	297	210	73 (9.8)
			no EOC	626	149	55	248	175	64 (10.3)
			EOC	125	30	10	50	35	9 (7.4)
			no complications	2,749 (78.5)	-	-	-	-	-
	ANC	6,500	complications	1,244 (19.1)	331	68	455	390	83 (6.7)
			no EOC	144	37	10	52	44	14 (9.4)
			EOC	1,100	294	58	403	346	70 (6.3)
			no complications	5,256 (80.9)	-	-	-	-	-
	**total**	**10,000**			**510**	**133**	**752**	**600**	**157**
**KSHI**	no ANC	1,500	complications	321 (21.4)	77	27	127	90	31 (9.6)
			no EOC	256	61	23	101	72	26 (10.3)
			EOC	65	16	5	26	19	5 (7.2)
			no complications	1,179 (78.6)	-	-	-	-	-
	ANC	8,500	complications	1,327 (15.6)	433	87	296	510	79 (6.0)
			no EOC	30	9	3	6	11	3 (10.9)
			EOC	1,297	424	84	290	499	76 (5.9)
			no complications	7,173 (84.4)	-	-	-	-	-
	**total**	**10,000**			**510**	**115**	**424**	**600**	**110**

SoC, standard of care; KSHI, Kwara state health insurance; ANC, antenatal care; EOC, essential obstetric care; PPH, post-partum heamorrhage; HTD, hypertensive disorders; OL, obstructed labour; n, number.

*death among complicated deliveries only.

In the KSHI scenario, the higher utilization of ANC and EOC translates into a lower number of sepsis and hypertensive disorder cases. The number of cases of post-partum haemorrhage and obstructed labour are the same in both scenarios because it was assumed that these complications have an incidence that is independent of previous access to healthcare; however, the outcomes of those complications do vary. Indeed, we observed fewer deaths in the KSHI scenario as opposed to the standard of care scenario, with an estimated total of 47 deaths averted per 10,000 deliveries.

In Table 3, we present the total cost for each scenario, the total number of DALYs, and the incremental cost-effectiveness ratio for the KSHI scenario compared to the standard of care for both the primary estimate and alternative base case assumptions.

**Table 3:** Cost-effectiveness of KSHI program (US$ 2012).

	total cost	total DALYs	cost per DALY	ICER, compared to SoC, mean	ICER, monte carlo simulation, median (2.5–97.5 percentile)
Primary estimate					
**SoC**	397,618	362,581	1.2	reference	reference
**KSHI**	755,690	370,305	2.0	46.4	49.1 (21.9–152.3)
Alternative base case scenarios					
**SoC 1**	517,976	364,007	1.4	reference	reference
**KSHI**	755,690	370,305	2.0	37.7	39.9 [16.9–157.5]
**SoC 2**	668,991	367,392	1.8	reference	reference
**KSHI**	755,690	370,305	2.0	29.8	29.6 [CS-191.1]
**SoC 3**	866,661	370,300	2.3	reference	reference
**KSHI**	755,690	370,305	2.0	CS	46.4 [CS-5,201.3]

SoC, standard of care; KSHI, Kwara state health insurance; DALY, disability-adjusted life year; ICER, incremental cost-effectiveness ratio; HIF, health insurance fund; CS, cost saving. Scenario 1 of the standard of care (SoC1) refers to an increased utilization of the standard of care clinics; scenario 2 of the standard of care (SoC2) refers to an increased cost and quality of care improvement in the standard of care clinics (ie access to EOC if delivery in a health facility and access to preventive treatment of hypertensive disorder complications if access to ANC); and scenario 3 of the standard of care (SoC3) refers to increased utilization, cost and quality of care improvement in the standard of care clinics.

We observe that the KSHI scenario has a higher total cost than the standard of care, which translates into a tangible benefit to patients in terms of a higher number of DALYs averted. The cost per DALY is small for both scenarios, reflecting the generally high ‘value for money’ of maternal health interventions. The ICER is considered very cost-effective compared to a willingness-to-pay threshold of one GDP per capita in Nigeria. In addition, we explored the cost-effectiveness acceptability curves to assess our estimates against a wide range of willingness-to-pay thresholds (from US$1 to 5,000). We found that from a willingness-to-pay threshold as low as US$200, it is very likely that KSHI care remains cost-effective compared to the base case (S1 File). We also present the 95% credible interval for the ICER to illustrate the amount of uncertainty around our point estimate. Under three alternative comparison scenarios, the KSHI care remains cost-effective. The first alternative scenario explores a base case scenario where the same utilization of maternal services is achieved compared to the KSHI scenario. The second set of assumptions model a base case where the costs are those estimated in the literature [48] and an increase in the quality of care is observed. The third set of assumptions explored an increase in utilization, costs and quality of care within the base case scenario. These alternative base case scenarios highlight an amount of uncertainty related to our conclusion that increases following changes in assumptions making the base case scenario similar to the intervention scenario.

### Budget impact

**Fig 2:**
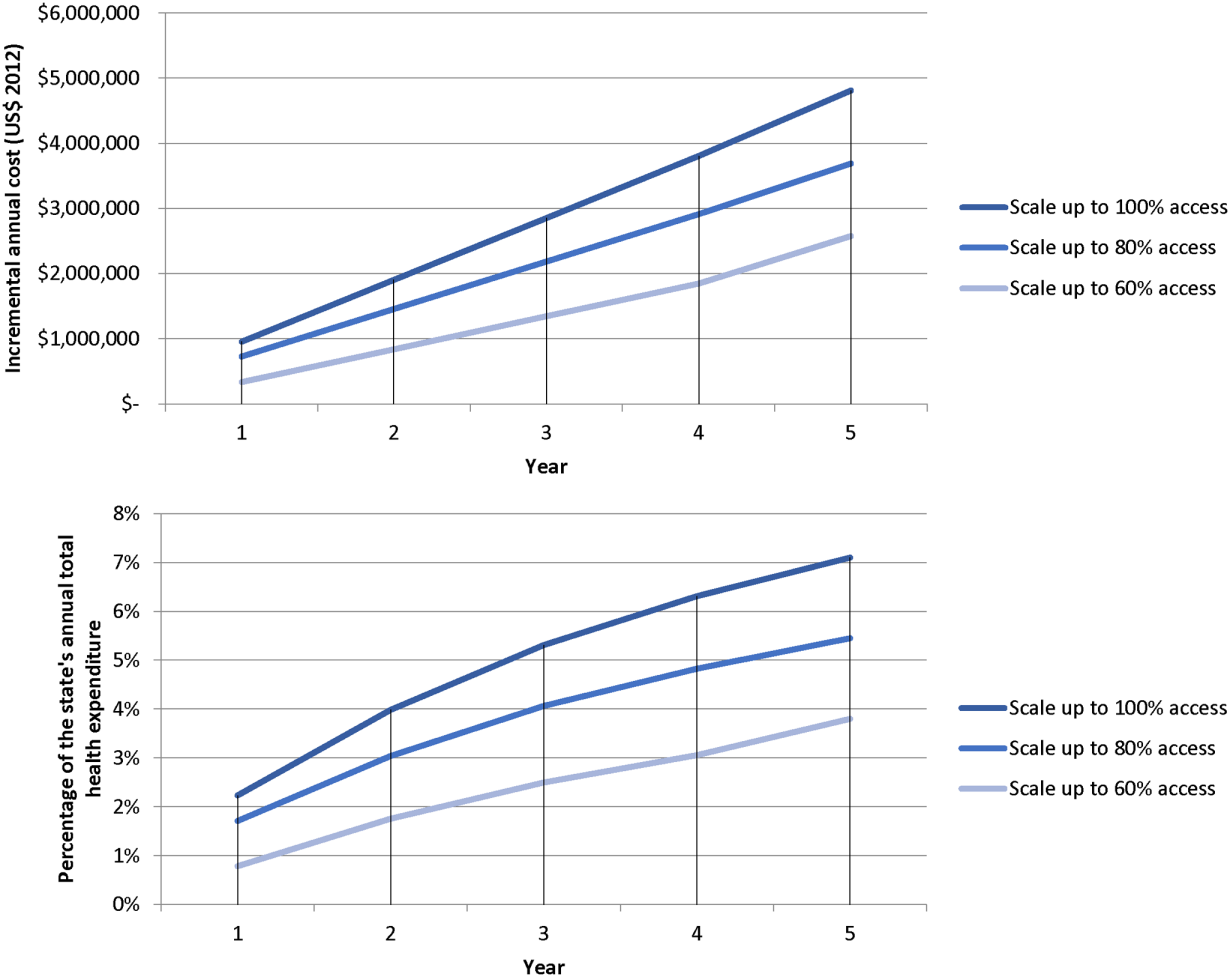
Projected incremental and relative annual cost of maternal care in Kwara state, Nigeria. Scale up scenarios refer to scenarios where the access to the insurance program is scaled up to 60, 80 or 100% of the population in need.

The annual cost of providing ANC and EOC to pregnant women in Kwara state was estimated to be $3,329,516 under base case assumptions of utilization and quality of care. Scaling up improvements in the quality of maternal care and its utilization is likely to increase the cost of maternal care in Kwara state. The magnitude of this increase depends on the level of scale up considered. The incremental annual cost by scale up scenario is shown in Fig 2a. Fig 2b shows the relative increase in state health expenditures required [43].

**Fig 3:**
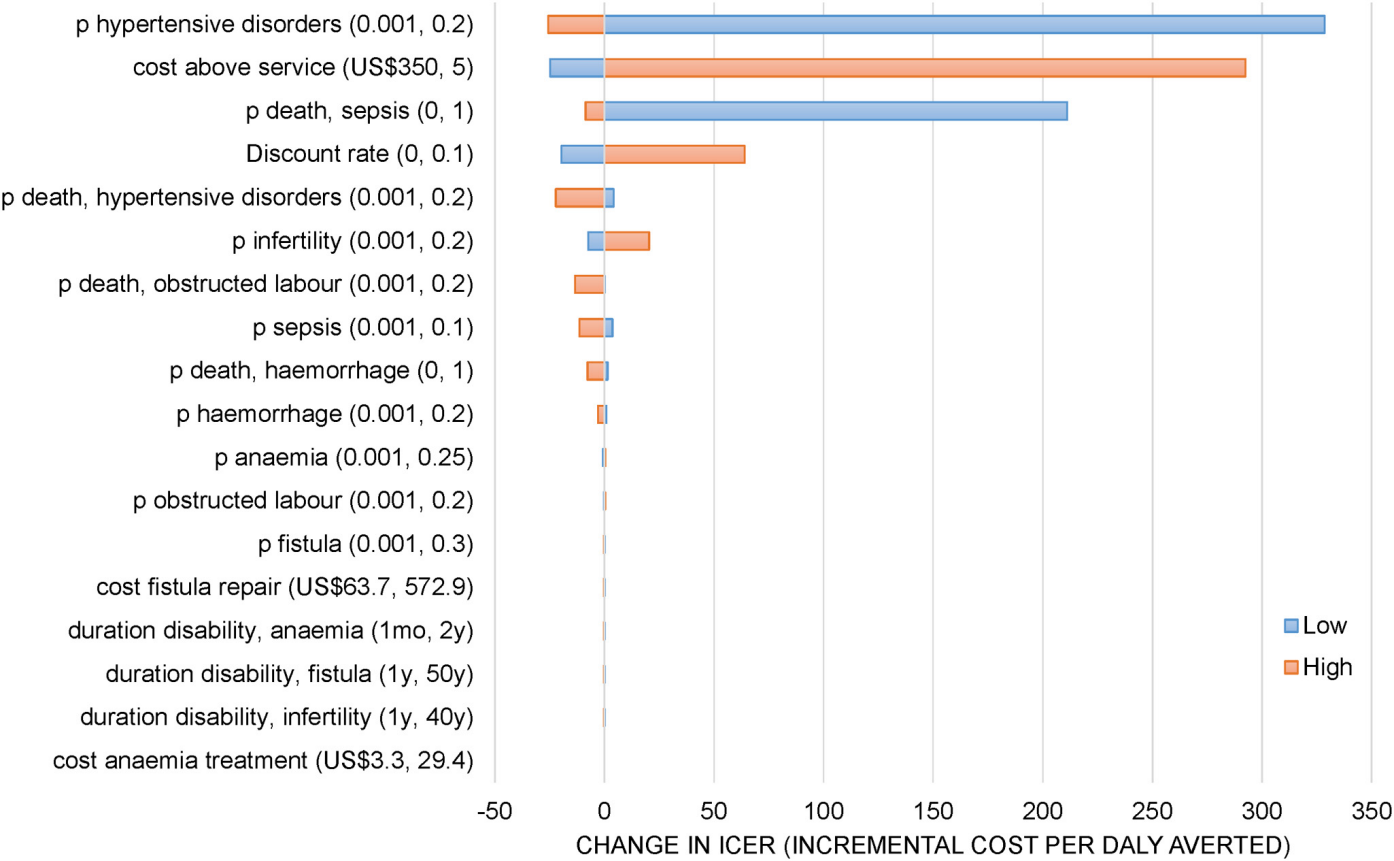
One-way sensitivity analysis comparing KSHI care vs standard of care. P, probability; y, year; mo, month; US$, US dollar. Blue bars represent the change in ICER when a parameter is varied to a lower value than the base case estimate. Red bars represent the change in ICER when a parameter is varied to a higher value than the base case estimate. All values for the parameters tested in this sensitivity analysis and the resulting ICERs are given in additional results (S1 File).

Finally, in Fig 3 we illustrate how sensitive our primary estimate is to extreme variation of parameter assumptions in a series of one-way sensitivity analyses (detailed results in S1 File).

We observe that our results are most sensitive to variations in the probability of hypertensive disorders, the above-service cost estimation, and the probability of death following a sepsis episode. To a lower degree, the results are sensitive to the discount rate used, the probability of death following a hypertensive disorder complication, and to the probability of infertility following a sepsis episode. However, the KSHI scenario remains cost-effective under all extreme variations considered.

## Discussion

Our results suggest that investing to improve both supply and demand for maternal health services is likely to have been a cost-effective intervention in rural Nigeria compared to the current standard of care. The extent of gains is dependent on a number of factors, including assumptions about the prevalence and severity of complications during delivery. While complications such as obstructed labour and post-partum haemorrhage themselves are generally not preventable, the KSHI program increased the likelihood that women access care in general and specifically for an emergency; thereby, improving the outcomes of complicated deliveries. Our conclusions remain stable under a wide range of sensitivity analyses and taking into account uncertainty in our parameter estimates. When we change our base case assumptions (to an increase in service utilization, an increased estimation of health service costs and quality of care, or both), the KSHI program remains likely to be considered cost-effective.

Although a WTP threshold of one GDP per capita is used as the currently recommended benchmark [39], there are important limitations with this decision rule for decision making [51]. These include the fact that even if an intervention might be considered cost-effective at this level, it might not be feasible within the current resource availability and use within the health financing structure. Furthermore, one GDP per capita might be considered too high a threshold for some countries [51]. The last point is particularly relevant in our study, as GDP per capita is a national measure, while we aim to inform decision making in one of Nigeria’s poorest states. We calculated the minimum threshold under which the intervention will likely not be considered cost-effective (US$200) to inform policy makers willing to vary the decision rule threshold in response to local constraints. Additionally, our budget impact analysis suggests that a minimum of 4% more investment in the State health care expenditure is needed to successfully scale up this program at a State level. This estimate is dependent on the model of care implemented. Furthermore, the feasibility of wide-scale expansion of the program is dependent on health system constraints such as a limited health workforce and health facility infrastructure, which were not included in the analysis. Finally, our analysis is based on regional health accounts up to 2005. Further collaborative work with Kwara State’s representatives to update these figures is ongoing looking at the financial space available to ensure the sustainability of the program transfer and scale up.

With regards to the cost estimates of the intervention, overall, ANC consultations were the main driver of the total costs for maternal care in the participating hospital, mainly due to the high service utilisation we observed. Complicated deliveries were estimated to be the most costly services, but due to their low number, they do not represent a significant part of the overall cost of maternal services at OOH. While an increase in ANC utilization in this community might lead to an increase in the total cost, it would likely be small and offset by reductions in complicated deliveries related to hypertensive disorders and sepsis.

Estimates of costs and cost-effectiveness studies for ANC and delivery interventions in Nigeria are scarce. However, our estimates are in accordance to those found in a recently published cost-effectiveness analysis of scale up of interventions to reduce pregnancy-related mortality [48].

This study has several limitations. The cost analysis presented uses data from several sources with reporting biases: in-clinic utilisation data might be incomplete and the act of observing consultations may alter the consultation process. To limit these biases, we aimed to spend significant periods of time in the clinics, during several visits, so that the researchers’ presence became more familiar and the behaviour of healthcare providers more normative. We also triangulated all information obtained and checked with local partners when there were any discrepancies. We generated two utilisation profiles to reflect the uncertainty in utilisation estimates. We included above-service program costs based partially around projections, increasing the uncertainty around these estimates. However, our results show that the KSHI intervention continues to be considered cost-effective, even when significantly increasing above-service program costs.

Because the intervention scenario consists of several interventions simultaneously targeting improvements to both the demand and supply side of health care utilization and because the impact evaluation empirically collected data on utilization and outcomes during program implementation to form the intervention scenario, we were unable to differentiate the impact of specific components of the program. However, we explored the impact of different base case assumptions in three scenarios during a scenario analysis. These scenarios looked at possible increases in cost and quality of standard of care services, utilization of these services, or both. Our conclusions remained robust to these changes. Yet, it is highly likely that given the (human) resource constraints in the region, costs of scaling up the program could be higher and/or health benefits lower than estimated. This would influence the cost-effectiveness of the scale up process. If possible, we recommend the assessment of the scaling up process to be conducted incrementally.

Finally, we limited our analysis to a healthcare provider perspective and were not able to assess the impact of the intervention on patient costs, specifically OOP expenditures related to maternal care. The costs incurred by patients in accessing health services affect patients’ health seeking behaviour, leading to poorer health outcomes, and could drive households into poverty [52]. There is a paucity of patient cost studies from Kwara State but experiences from other settings have shown that even where services are provided free or are subsidized at the point of care, transport costs, and income loss can impede access to care or lead to catastrophic expenditure [53]. Through the KSHI program, individual beneficiaries, who live on less than US$1.5 a day, are enrolled on an annual basis, paying a premium of approximately US$2 per person per year [54]. The scheme’s beneficiaries do not pay OOP for services at the point of care; indeed a 52% total reduction in OOP spending on all healthcare has been attributed to the KSHI program [54]. Our analysis from a health service perspective is therefore likely to underestimate the program cost-effectiveness by not including benefits from any financial risk protection impact of the KSHI program. However, uptake and renewal of membership is limited by household ability to pay the annual insurance co-premium [55], which might threaten the expansion and sustainability of the program. Particular attention when implementing and expanding the program should be paid to the design of supply side of the intervention to avoid any issues of inequity affecting uninsured populations. Indeed, previous research highlighted the possible negative effects on those who did not enrol in the insurance (in terms of a decrease in healthcare utilization) [56].

## Conclusion

An intervention aiming to improve utilization and quality of maternal care, such as the KSHI program, is likely to be a cost-effective investment compared to current standard of care, even when including significant costs for provision of technical assistance. Policy makers should consider sustaining similar state-wide initiatives to reduce maternal mortality, being aware that the budgets available for healthcare must increase to avoid the annual insurance premiums that are a barrier for the poor on the road towards Universal Health Coverage.

## Supporting Information

S1 File Data, model description and additional results. (DOCX)
