# Supplementary material for: Improving Maternal Care through a State-Wide Health Insurance Program: A Cost and Cost-Effectiveness Study in Rural Nigeria
Source: PLoS One. 2015 Sep 28;10(9):e0139048. doi: 10.1371/journal.pone.0139048 (PMC4587550; doi:10.1371/journal.pone.0139048)
Supplement: S1 File — (DOCX) [file pone.0139048.s001.docx]

Improving maternal care through a state-wide health insurance program: a cost and cost-effectiveness study in rural Nigeria

G. B. Gomez, N. Foster, D. Brals, H. E. Nelissen, O. A. Bolarinwa, M. E. Hendriks, A. C. Boers, D. van Eck, N. Rosendaal, P. Adenusi, K. Agbede, T. M. Akande, M. Boele van Hensbroek, F. W. Wit, C. A. Hankins, C. Schultsz

SUPPORTING INFORMATION (S1_SUPPORTING INFORMATION). DATA, MODEL DESCRIPTION, AND ADDITIONAL RESULTS

v. AUGUST 2015

Table of Contents

[Comparators 3](#_Toc426965533)

[Current standard of care 3](#_Toc426965534)

[Services 3](#_Toc426965535)

[Complications during delivery 4](#_Toc426965536)

[Kwara State health insurance (KSHI) scenario 7](#_Toc426965537)

[Services 7](#_Toc426965538)

[Complications during delivery 7](#_Toc426965539)

[Outcome measures 8](#_Toc426965540)

[Maternal deaths averted 8](#_Toc426965541)

[Disability adjusted life years averted 8](#_Toc426965542)

[Costs 10](#_Toc426965543)

[KSHI scenario 10](#_Toc426965544)

[Setting 10](#_Toc426965545)

[Service cost identification and measurement 10](#_Toc426965546)

[Overview of service costs and utilization data 12](#_Toc426965547)

[Above-service costs 14](#_Toc426965548)

[Reference scenario 16](#_Toc426965549)

[Overview of costs 16](#_Toc426965550)

[Sensitivity analysis 17](#_Toc426965551)

[Additional results 19](#_Toc426965552)

[References 21](#_Toc426965553)

# Comparators

## Current standard of care

The current standard of care in rural Nigeria (base case) was characterized in two dimensions: utilization and quality of care. The parameters and assumptions described below were derived from nationally or regionally representative surveys, maternal health audits, and data collection as part of the baseline survey in 2009 of the general impact evaluation of the KSHI program (1).

### Services

#### Antenatal care

Antenatal care (ANC), defined as the regular medical and nursing care recommended for women during pregnancy, is important for the prevention of mortality and morbidity in both the mother and her child (2). The ANC policy in Nigeria follows the World Health Organization (WHO) approach recommending at least four ANC visits for women without complications (3,4). Fifty-one percent of women surveyed in the Demographic Health Survey (DHS) of 2013 reported having at least four ANC visits, the median number of months pregnant at first visit was five (5). These estimates are higher than those found in the literature (25-36%) (6)(7). However, we based our estimated proportion of women attending ANC, on data available for the indicator ‘at least one ANC visit during pregnancy’ from the observed data from the KSHI program impact evaluation survey (0.64, standard deviation 0.48) (1) and the estimate reported in the Nigerian DHS, 2013 (60.6% of women had at least one ANC visit with a skilled provider, most commonly from a nurse or midwife (35.3%) or a doctor (25.3%)) and the National HIV & AIDS and Reproductive Health Survey (NARHS), 2012 (66.2% for the North Central region) (5,8). We chose this indicator of utilization (at least one ANC visit during pregnancy) because a model with a lower number of outcomes, while providing the most conservative estimate for the base case (9).

We defined the quality of standard ANC services by its components as reported in both the NARHS 2012 and DHS 2013 for the North Central region or Kwara State respectively:

- Weight taken: 90.7% (8)
- Prescription of iron tablets: 96.1% (5)
- Prescription intestinal parasite drugs: 22.9% (5)
- Information on pregnancy complications: 89.8% (5), 57.2% (8)
- Blood pressure measurement: 98.0% (5), 87.9% (8)
- Urine sample collected: 96.8% (5), 82.3% (8)
- Blood specimens collected: 96.3% (5), 77.7% (8)
- Tetanus toxoid vaccination: 80.4% (5), 80.7% (8)
- Tested for HIV during ANC: 45.4% (8)

#### Delivery care

Delivery care is defined as care during the delivery by a healthcare professional. In the DHS 2013 survey (5), it was reported that, nationally, about one-third of births (35.8%) occur in health facilities (22.6% in public sector and 13.2% in private sector facilities). Home births are more common in rural (76.9%) compared to urban areas (37.4%). For those women receiving no ANC, it was reported that 95.1% delivered at home. In Kwara State, 76.7% of participants reported delivering at a health facility, this included both primary health care and hospitals (5). In the 2012 NARHS, 71.8% reported delivering at a health facility (with 61.3% delivering at a hospital either public or private) (8). This estimate is higher than the observed percentage of women delivering in hospital in the population-based surveys of the impact evaluation of the KSHI program (48% in 2009 pre-intervention) (10).

The most cited reasons for not delivering in a health facility in Kwara State were that the child was born suddenly and there was no time to reach the facility (41%). This finding may highlight the need for improvements in the utilization and quality of ANC received, e.g. better estimated expected date of delivery. Other reasons cited were: cost (10.8%), facility is too far/no transportation available (13.4%), and it was not deemed necessary (21.2%) (5).

#### Essential obstetric care

Essential obstetric care (EOC) is defined as care including the ability to administrate parenteral antibiotics, parenteral oxytocic drugs, and parenteral anticonvulsants for pre-eclampsia and eclampsia; ability to perform manual removal of placenta and of retained products; ability to perform assisted vaginal delivery, surgery (C-section), and blood transfusions(11). Access to EOC depends on the location of the delivery. For those women delivering at a healthcare facility (hospital), a utilization of EOC was assumed to be 90-95% (12). For those women delivering either at home or in a primary care facility, 13.6% were assumed to access EOC based in a recent audit (13) in Kwara State.

### Complications during delivery

We considered four complications during delivery in this analysis: post-partum haemorrhage, obstructed labour, hypertensive disorder, and sepsis. We summarised the estimated probability of having a specific complication in table S1. In tables S2 and S3, we present mortality and morbidity (14) parameters by complication. The assumptions leading to these parameters are described below.

#### Post-partum haemorrhage

Globally, the prevalence of post-partum haemorrhage, defined as an excessive bleeding (more than 1000 mL) from the genital tract (15), has been estimated at approximately 6% of all deliveries (16). However, significant heterogeneity among regions was found in this systematic review. Looking at regional variability, a recent systematic review estimated the prevalence of post-partum haemorrhage at 5.1% (95%CI 0.3-15.3) in Africa (17). Among post-partum haemorrhage events, the case fatality rate was estimated at 1%; and among those surviving, an estimated 12% suffer from severe anaemia, defined as a blood haemoglobine level of less than 9mg per dL (18). For women accessing EOC, we assumed a risk reduction of 0.34 (95%CI 0.14-0.87) in the risk of post-partum haemorrhage and of 0.50 (95%CI 0.30-0.83) in the risk of developing severe anaemia (19).

#### Obstructed labour

The prevalence of obstructed labour, defined as labour in which progress is arrested by mechanical factors (15), has been reported to be approximately 6% in the African region including Nigeria (20). Among neglected obstructed labour events, the case fatality rate was estimated at 0.7% (18); among those surviving, an estimated 14% of all cases of obstructed labour may continue to suffer from recto-vaginal or vesico-vaginal fistula, defined as a communication between the vaginal wall and the bladder/rectum resulting from obstructed labour (20). We assumed that women die or develop a fistula following neglected obstructed labour only. Neglected obstructed labour will occur in those women not accessing EOC. For those women accessing EOC, we estimated that in 90% of cases of obstructed labour, a caesarean section is carried out, and in the remaining 10% an instrumental delivery (18). The frequency of caesarean sections is the same for women attending ANC and those not doing so. Evidence shows that external cephalic version of breech presentations at term reduces the rate of caesarean section and of breech delivery; however it does not reduce this rate if the procedure is done before term and these cases represent a very small proportion of the overall burden of obstructed labour (2).

#### Hypertensive disorders

Hypertensive disorders are defined as a group of conditions where there is raised blood pressure with proteinuria (15) during the pregnancy. Pre-eclampsia and eclampsia (eclampsia being a consequence of pre-eclampsia) are the most serious consequences for the mother and child. The long-term sequelae of both pre-eclampsia and eclampsia are not well evaluated, and the burden of hypertensive disorders stems mainly from deaths (18). The incidence of pre-eclampsia and eclampsia vary by region, and for the African region it has been estimated at 5.6% (95%CI 3.6–11.3) and 2.9% (95%CI 1.4–7.4), respectively (21). Case fatality rate due to hypertensive disorders have been reported to be 8.3% (21). Magnesium sulphate reduces the risk of developing eclampsia in pregnancy (risk ratio 0.41, 95%CI 0.29-0.58) (22). We assumed magnesium sulphate was only available to 30% of women accessing ANC (2,23,24).

#### Puerperal sepsis

The WHO defines sepsis as an ‘infection of the genital tract occurring at any time between the onset of rupture of membranes or labour and the 42^nd^ day postpartum in which fever and one or more of the following are present: pelvic pain, abnormal vaginal discharge, abnormal smell/foul odour of discharge and delay in the rate of reduction of the size of the uterus’ (25). In Nigeria, the prevalence of puerperal sepsis has been estimated at 1.7% (26). Women delivering in a facility have half the risk of sepsis compared to those delivering at home (27). The main non-fatal consequence of sepsis is secondary infertility resulting from tubal occlusion (18), defined as the failure to conceive again after a previous conception (15). It has been estimated that approximately 7.5% of women surviving a sepsis episode are infertile as a result (18,27).

Table S1 – Probability of event: complications during delivery.

| **Complication** | **Comments** | **Model input** | **References** |
| --- | --- | --- | --- |
| Post-partum haemorrhage | mean (SD) | 0.051 (0.038) | (16,17,28) |
| Obstructed labour | mean (min-max) | 0.06 (0.03-0.09) | (12,20) |
| Hypertensive disorders | mean (95%CI) | 0.085 (0.187-0.05) | (21) |
| Sepsis | mean (min-max) | 0.017 (0.01-0.03) | (26) |

SD, standard deviation; min, minimum; max, maximum; CI, confidence interval.

Table S2 – Probability of event: morbidity.

| **Complication** | **Morbidity** | **Model input** | **References** |
| --- | --- | --- | --- |
| Post-partum haemorrhage | Anaemia, mean (min-max) | 0.12 (0.10-0.14) | (18) |
| Obstructed labour | Fistula, mean (min-max) | 0.14 (0.13-0.16) | (20) |
| Sepsis | Infertility, min-max | 0.05-0.1 | (18,27) |

min, minimum; max, maximum.

Table S3 – Probability of event: case fatality rate by complications.

| **Complication** | **Comments** | **Model input** | **References** |
| --- | --- | --- | --- |
| Post-partum haemorrhage | per 100 deliveries, min-max | 2.8-27.3 | (28) |
| Obstructed labour | per 100 deliveries, mean (min-max) | 0.7 (0.6-0.8) | (18,29) |
| Hypertensive disorders | Per 100 pregnancies, mean (min-max) | 8.3 (5-10) | (21) |
| Sepsis | per 100 deliveries, min-max | 0-72.7 | (27,28) |

min, minimum; max, maximum.

Finally, we aimed to validate our standard of care scenario by comparing the number of deaths by complication predicted by the model to those reported in the literature. Our model seems to be a good fit predicting approximately 45 deaths per 10000 pregnancies due to haemorrhage, 49 to sepsis, and 2 to obstructed labour. This corresponds to the same order of magnitude as reported in a Nigerian cohort recently (30).

## Kwara State health insurance (KSHI) scenario

### Services

#### Antenatal care

The proportion of insured women utilizing ANC during their last pregnancy was reported at 0.87 (standard deviation 0.34) in our household survey (1). ANC services provided in OOH are described in detail in table S8 (ANC cost).

#### Delivery care

The proportion of insured women delivering in hospital increased to 70% in our household survey in 2011 and 69% in 2013 (10). We assumed the same ratio for the deliveries hospital:home or other primary health facility as in the base case.

#### Essential obstetric care

In this scenario, access to EOC also depends on the location of the delivery. For those women delivering at home or at primary care facilities, we made the same assumptions as in the base care. Of those women delivering in OOH (our comparator setting), 100% have access to EOC, as this is part of the insurance package and available at this hospital.

### Complications during delivery

We assumed the same estimated probability of having a specific complication, mortality and morbidity to be dependent on access to care, as in the standard of care scenario (tables S1, S2, and S3). These proportions are in accordance to those observed in OOH (e.g. we observed 23 episodes of post-partum haemorrhage in 2011-2012, 4% of all deliveries). The only exception was that the proportion of women developing complications due to hypertensive disorders was lower in the KSHI scenario compared to the standard of care scenario, because magnesium sulphate was available to all women accessing ANC at OOH for prevention of hypertensive disorders’ complications.

# Outcome measures

We present two measures of effectiveness in this study: maternal deaths averted and disability-adjusted life years (DALYs) averted.

## Maternal deaths averted

Deaths averted are calculated incrementally from the difference between the total number of women dying in the standard of care scenario and the KSHI care scenario.

## Disability adjusted life years averted

Clinical outcomes (such as mortality and morbidities) were translated into DALYs, and compared incrementally across the two scenarios.

We present DALYs instead of QALYs for two main reasons: DALYs are useful when comparing across diseases. We aim for this piece of work to feed into a broader portfolio of work to be presented to policy makers in Nigeria looking at a number of conditions. Secondly, there is no data on quality of life questionnaires for the complications in this model available locally.

Total number of DALYs averted are calculated as the sum of the number of years of life lost (YLL) and the number of years lost due to disability (YLD) using published methods (31). These calculations include local life expectancy and duration of disability. Duration of disability was calculated using the following assumptions:

- for anaemia: we assumed an average duration of six months because no discharge follow-up information was available to assess the sequalae, if any, of this disability and this is the recommended duration of treatment for severe anaemia (32). However, we tested the impact of this assumption on our results via a one-way sensitivity analysis.
- for recto-vaginal fistula: it is estimated that in Nigeria, approximately 20,000 new obstetric fistulae occur every year. Due to the current backlog in surgery, it is estimated that only 2,500 cases have access to reconstructive surgery annually. Therefore, assuming the average age of a pregnant woman from the DHS at 27.6 years old (33), a local life expectancy at this age of 40.4 years (34) and that only 10% of prevalent cases get reconstructive surgery with an average waiting period of 1.8y (35) while the rest will endure a lifelong disability, the average duration of disability was estimated to be 36.6 years. These assumptions have limitations. For instance, the risk for fistula is highest among the youngest women, especially after delivering their first baby. In this analysis, we opted for an average age at pregnancy because we have little information on the distribution of fistula incidence by age in Nigeria and because the assumptions on DALYs are the same for both scenarios, limiting the impact of this simplification on our results. However, we performed a sensitivity analysis on this particular assumption.
- for infertility: we assumed a duration of disability over the average national reproductive span (49 years) from the average age of a pregnant woman (27.6 years old) (33), that is 21.4 years. However, we performed a sensitivity analysis on the duration of disability to assess the impact of this assumption on our results.

We present DALYs in the main results using a discount rate of 3% and excluding age-weighting as recommended in the latest Global Burden of Disease Study 2010 (14). However, in sensitivity analysis we varied the discount rate between 0 and 10%. Tables S4 and S5 show the assumptions for the calculation of DALYs and the DALYs averted by mortality and morbidity.

Table S4 – DALY assumptions.

|  | **Age-weighting modulation constant** | **Age of onset of disease**  **(years)** | **Disability duration (years)** | **Age at death**  **(years)** | **Standard life expectancy at age of death (years)** | **Disability weight** | **Discount rate** |
| --- | --- | --- | --- | --- | --- | --- | --- |
| Death | 0.00 | 27.57 | 0.00 | 27.57 | 40.44 | 0.00 | 0.03 (0-0.1) |
| Disability: anaemia | 0.00 | 27.57 | 0.5 | 68.01 | 0.00 | 0.16 | 0.03 (0-0.1) |
| Disability: fistula | 0.00 | 27.57 | 36.57 | 68.01 | 0.00 | 0.49 | 0.03 (0-0.1) |
| Disability: infertility | 0.00 | 27.57 | 21.43 | 68.01 | 0.00 | 0.01 | 0.03 (0-0.1) |

Table S5 – Total DALYs averted.

| 1. **Discounted (discount rate: 0.03)** | | | |
| --- | --- | --- | --- |
|  | DALYs due to YLD | DALYs due to YLL | Total DALYs averted |
| Death | 0.00 | 23.42 | 23.42 |
| Disability: anaemia | 0.08 | 0.00 | 0.08 |
| Disability: fistula | 10.93 | 0.00 | 10.93 |
| Disability: infertility | 0.09 | 0.00 | 0.09 |
| 1. **Not discounted (discount rate: 0)** | | | |
|  | DALYs due to YLD | DALYs due to YLL | Total DALYs averted |
| Death | 0.00 | 40.44 | 40.44 |
| Disability: anaemia | 0.08 | 0.00 | 0.08 |
| Disability: fistula | 17.99 | 0.00 | 17.99 |
| Disability: infertility | 0.13 | 0.00 | 0.13 |

| 1. **Discounted (discount rate: 0.1)** | | | |
| --- | --- | --- | --- |
|  | DALYs due to YLD | DALYs due to YLL | Total DALYs averted |
| Death | 0.00 | 9.82 | 9.82 |
| Disability: anaemia | 0.08 | 0.00 | 0.08 |
| Disability: fistula | 4.79 | 0.00 | 4.79 |
| Disability: infertility | 0.05 | 0.00 | 0.05 |

DALY, disability-adjusted life years; YLD, years lived with disability; YLL, years of life lost

# Costs

## KSHI scenario

### Setting

The Ogo Oluwa Hospital (OOH) in Kwara State, rural Nigeria is a private hospital participating in the Kwara state health insurance (KSHI) program. Kwara State is part of the North Central region of Nigeria and has a population of 2,371,089 (2006 estimate)(36). The OOH serves the community of Bacita, part of Edu local government area (population estimated: 201,642 in 2006(37)). The hospital provides antenatal and perinatal care as well as essential obstetric care, including caesarean sections for complicated deliveries. A total of 50 employees [three doctors, nine nurses and community health education workers, 11 ward assistants, eight laboratory staff members (one scientist, one technician and six assistants), four pharmacy staff members, and 15 administrative staff members] were working in OOH during 2011-2012. The average number of patients enrolled in the SHIS registered in OOH was 9,738 for the period 2010-2011. These patients represented over 95% of the total patient population of OOH (personal communication, medical director OOH). Further description of the setting is given in (38).

### Service cost identification and measurement

A costing exercise was undertaken from a healthcare provider's perspective using a micro-costing approach (39)(40)(41). To estimate the cost of ANC and delivery care, first we identified all functional components of the program within a clinical pathway defined as the succession of activities a pregnant woman would be exposed to in the hospital from confirmation of pregnancy to delivery and post-partum care. The clinical pathway was built from interviews with clinicians and observations of practice.

In this pathway, ANC includes: an initial health education group session, consultations, drugs prescribed and obtained from the pharmacy (folic acid, multivitamin, iron supplement, vitamin C, vitamin B complex, paracetamol, and tetanus toxoid), and tests performed at OOH (blood group, packed cell volume, HIV screening, hepatitis screening, syphilis screening, urine analysis (dipstick), microalbuminuria, random blood glucose, Giemsa stain for malaria parasites, and ultrasound). For women presenting to the hospital for delivery, we defined an uncomplicated delivery to include: an examination upon presentation (history taking, physical examination, maternal blood pressure check, and foetal heart rate), monitoring activities during delivery, and the administration of an intravenous fluid infusion. After delivery care following an uncomplicated delivery includes oxytocin/ergometrine to prevent post-partum haemorrhage and the prescription of the following drugs and supplements: folic acid, multivitamin, iron supplements, vitamin C, and paracetamol. No tests were observed during uncomplicated deliveries, however the use of certain antibiotics (e.g. ampicillin/cloxacillin, metronidazole, gentamicin) was observed and were included in the unit cost. A complicated delivery is defined as a delivery requiring EOC. In addition to the activities observed during uncomplicated deliveries, we observed that complicated deliveries also include more personnel time, the use of specialized medical equipment, drugs for C-sections. Additional tests that were observed during the complicated deliveries only in addition to the initial examination were blood group, packed cell volume, HIV screening, hepatitis screening, and random blood glucose. We acknowledge that these are not tests particular of any complication during delivery, however they were only performed during complicated deliveries in OOH. We also observed hospital stays for complicated deliveries that was added to the unit cost.

For each of the defined activities, we then identified inputs required: capital and recurrent costs classified in two categories: direct and indirect costs. Direct costs information collected included consumables (utilisation and unit price), drugs (prescriptions and unit price), medical equipment used for tests and during delivery, hospital bed utilisation during ANC consultations or in-patient delivery (utilisation, price, and years of use), and staff time (staff category, working time).

Data collection activities took place from June 2011 to September 2012. Data included in the analysis covered the period from October 2011 to September 2012. Table S6 shows the schedule of all data collection activities. We collected data on resource utilisation through the in-clinic reporting system (where the healthcare professionals record tests, drugs and examinations undergone by each patient during a specific visit), activity logs (where healthcare professionals record the activities and duration of activities during specific weeks), and interviews of clinicians. We interviewed the medical director and one doctor, three nurses, one community health education workers, the head of the laboratory, and one pharmacist. We also recorded a total of 57 observations of practice (where the researcher observed activities recording both type and duration): 45 out-patient ANC visits, four out-patient deliveries, and eight in-patient deliveries. Observations of ANC consultations took place during the ANC clinic day (OOH provides a dedicated day per week for ANC consultations) and were repeated twice. Deliveries were observed as they presented during the observation weeks. Finally, we compared the observation data on deliveries with the in-clinic maternal log book for three periods: January, March and June 2012.

Table S6 – Cost data collection activities.

|  | **2011** | | | | | | | **2012** | | | | | | | | | |
| --- | --- | --- | --- | --- | --- | --- | --- | --- | --- | --- | --- | --- | --- | --- | --- | --- | --- |
|  | J | J | A | S | O | N | D | J | F | M | A | M | J | J | A | S | O |
| Hospital expenditure sheets |  |  |  |  |  |  |  |  |  |  |  |  |  |  |  |  |  |
| Price data |  |  |  |  |  |  |  |  |  |  |  |  |  |  |  |  |  |
| ·         Equipment |  |  |  |  |  |  |  |  |  |  |  |  |  |  |  |  |  |
| ·         Consumables |  |  |  |  |  |  |  |  |  |  |  |  |  |  |  |  |  |
| ·         Drugs |  |  |  |  |  |  |  |  |  |  |  |  |  |  |  |  |  |
| Activity logs |  |  |  |  |  |  |  |  |  |  |  |  |  |  |  |  |  |
| Interviews |  |  |  |  |  |  |  |  |  |  |  |  |  |  |  |  |  |
| Observations |  |  |  |  |  |  |  |  |  |  |  |  |  |  |  |  |  |
| Maternal care logbook |  |  |  |  |  |  |  |  |  |  |  |  |  |  |  |  |  |

Note: Green denotes the period for which data was included in the cost analysis.

Indirect costs were those associated with overheads, building, and general equipment utilisation (not medical equipment directly employed during the clinical activities identified above). All indirect cost information was sourced from expenditures sheets provided by the hospital. We calculated a mark-up percentage that reflects the ratio of direct:indirect costs at a hospital level. This mark-up was added to the direct costs to calculate total costs.

To reduce the likelihood of missing any items and improve the validity of our estimates, we triangulated resource utilisation information from activity logs, interviews and observations with in-clinic records from the KSHI program on services and tests provided at OOH. We also verified the cost of drugs and consumables sourced from the suppliers with central Health Insurance Fund program records and international databases (i.e. <http://www.supply.unicef.dk/catalogue/index.htm>). All costs are presented in 2012US$. Preliminary results were presented to local staff members and discrepancies and/or outliers were verified. Finally, we compared our final cost estimates to other estimates available in the literature for Nigeria.

### Overview of service costs and utilization data

Service utilization in OOH is presented in table S7 for the period October 2011 to September 2012.

Table S7 - Service utilisation.

|  | **N** |
| --- | --- |
| Pregnancies | 1,563 |
| Total ANC visits | 4,852 |
| Total deliveries uncomplicated | 500 |
| Total deliveries complicated | 118 |

The unit costs per pregnancy for ANC, or per delivery for uncomplicated and complicated deliveries are described in table S8.

Table S8 – Detailed costs and utilisation of services.

|  | |  | Utilisation^a^ | Unit cost (2012US$) |
| --- | --- | --- | --- | --- |
| **Cost of ANC/pregnancy (total, including indirect costs)** | | |  | **12.4-61.5** |
| Cost of ANC/visit (total, including indirect costs) | | |  | 4.0-12.5 |
| Cost of ANC/pregnancy (direct costs) | | |  | 7.03-34.89 |
| Cost of ANC/visit (direct costs) | | |  | 2.3-7.1 |
| Consultation | | | 3.1-4.94^b^ | 0.51 |
| Health education group session/pregnancy | | | 1 | 0.01 |
| Tests/pregnancy | Packed Cell Volume | | 0.60-1 | 0.27 |
|  | HIV screening | | 0.63-1 | 2.15 |
|  | Hepatitis screening | | 0.32-1 | 0.99 |
|  | Blood group test | | 0.45-1 | 0.49 |
|  | Rapid treponemal point of care test | | 0.31-1 | 0.77 |
|  | Urine dipstick analysis | | 0.63-1 | 0.41 |
|  | Microalbuminuria test | | 0.02-1 | 3.56 |
|  | Random blood glucose test | | 0.29-1 | 0.22 |
|  | Giemsa stain for malaria parasites | | 0.05-1 | 1.58 |
|  | Ultrasound | | 0.06-1.5 | 11.50 |
| Prescriptions/pregnancy | Multivitamins | | 2.67-4.94 | 0.25 |
|  | Folic acid supplement | | 2.62-4.94 | 0.03 |
|  | Iron supplement | | 2.59-4.94 | 0.12 |
|  | Vitamin C supplement | | 0.19-4.94 | 0.09 |
|  | Vitamin B complex supplement | | 0.09-4.94 | 0.06 |
|  | Paracetamol | | 2.10-4.94 | 0.07 |
|  | Tetanus toxoid^c^ | | 0.24-0.61 | 0.05 |
| **Cost of care/uncomplicated delivery (total, including indirect costs)** | | |  | **9.65-27.2** |
| Cost of care/uncomplicated delivery (direct costs) | | |  | 5.47-15.42 |
| Tests/delivery | Packed Cell Volume | | 0.05-1 | 0.27 |
|  | HIV screening | | 0.04-1 | 2.15 |
|  | Hepatitis screening | | 0.03-1 | 0.99 |
|  | Blood group test | | 0.03-1 | 0.49 |
| Drugs/delivery | Ergotamine | | 0.01-1 | 0.22 |
|  | Oxytocin | | 0-0.3 | 0.15 |
|  | 5% Dextrose water | | 0-0.7 | 0.63 |
|  | Folic acid | | 0.81-1 | 0.03 |
|  | Multivitamin | | 0.83-1 | 0.25 |
|  | Iron supplement | | 0.79-1 | 0.12 |
|  | Vitamin C | | 0.12-1 | 0.05 |
|  | Ampicillin/cloxacillin (500mg) | | 0.06-1 | 5.69 |
|  | Metronidazole (Flagyl tabs 400mg) | | 0.07-0.3 | 0.01 |
|  | Paracetamol | | 0.69-1 | 0.03 |
|  | Gentamicin | | 0.01-0.3 | 0.05 |
| **Cost of care/complicated delivery (total, including indirect costs)** | | |  | **46.7-53.3** |
| Cost of care/complicated delivery care (direct costs) | | |  | 26.5-30.2 |
| Tests/delivery | Packed Cell Volume | | 0.56-1 | 0.27 |
|  | HIV screening | | 0.37-1 | 2.15 |
|  | Hepatitis screening | | 0.2-1 | 0.99 |
|  | Blood group test | | 0.29-1 | 0.49 |
|  | Urine analysis | | 0.30-1 | 0.41 |
|  | Blood glucose | | 0-0.1 | 0.22 |
| Drugs/delivery | Egomterin | | 0-0.03 | 0.22 |
|  | N/saline | | 0-1 | 0.63 |
|  | Atropine | | 0-1 | 0.15 |
|  | Ketamine | | 0-1 | 1.77 |
|  | Valium | | 0-1 | 0.15 |
|  | Gentamicin | | 0-0.03 | 0.05 |
|  | Ampicillin/cloxacillin (500mg) | | 0-0.3 | 5.69 |
|  | Folic acid | | 0.7-1 | 0.03 |
|  | Iron supplement | | 0.49-1 | 0.12 |
|  | Ciproflaxcin | | 0.04-1 | 0.09 |
|  | Vitamin C | | 0.31-1 | 0.05 |
|  | Paracetamol | | 0.57-1 | 0.03 |
|  | Metronidazole (Flagyl tabs 400mg) | | 0.03-1 | 0.01 |

Notes:

^a^ Utilisation : value ranges correspond to in-clinic data and the data collected through observations and interviews

^b^ number of visits [min-max]

^c^ tetanus toxoid is given a maximum of once per pregnancy

These cost estimates were robust to the changes in cost assumptions tested (Table S9). The largest variation was observed in the unit cost of complicated delivery when varying the assumptions made in the calculation of indirect costs (large variation in the mark-up percentage).

Table S9 - Sensitivity analysis on costs assumptions.

|  |  |  | **Unit cost (US$, low-high)** | | |
| --- | --- | --- | --- | --- | --- |
| Variable |  |  | ANC | Uncomplicated delivery | Complicated delivery |
| Mark-up | Baseline value | 76% | **4.0-12.5** | **9.65-27.2** | **46.7-53.3** |
|  | SA range | 50% | 3.4-10.6 | 8.2-23.1 | 39.7-45.3 |
|  |  | 90% | 4.3-13.4 | 10.4-29.3 | 50.3-57.4 |
| Staff time | Baseline value | reference | **4.0-12.5** | **9.65-27.2** | **46.7-53.3** |
|  | SA range | -20% | 3.9-12.3 | 8.8-26.1 | 44.9-51.4 |
|  |  | +20% | 4.1-12.7 | 10.5-28.3 | 48.4-55.2 |
| Medical equipment | Baseline value | reference | **4.0-12.5** | **9.65-27.2** | **46.7-53.3** |
|  | SA range | -20% | 3.8-11.4 | 9.6-26.6 | 43.5-49.7 |
|  |  | +20% | 4.2-13.5 | 9.7-27.8 | 49.9-56.8 |

### Above-service costs

The Health Insurance Fund (HIF) was founded in 2006 and received a grant from the Dutch Ministry of Foreign Affairs. PharmAccess Foundation is the implementing partner of the Health Insurance Fund and the technical partner in the Kwara State Health Insurance program.

In order to account for all costs related to the operational activities of the insurer and program management at PharmAccess level, we divided the ‘above-service’ costs into three components:

1. Costs related to operational activities at insurer Hygeia Community Health Care (HCHC) level, including costs of scaling up the program;
2. Costs related to the upgrading of the health care providers in the network;
3. Costs related to technical assistance delivered by PharmAccess Foundation.

The first component covers the operational costs at HCHC level. This includes costs of marketing activities and administration of the insurance product. For these costs, HCHC receives an income from the insurance premium paid by enrolees. Since sufficient scale has not been achieved, additional investment from the HIF is required to cover all expenditures. Once scale has been achieved, it is expected that HCHC will no longer receive the additional income. In this analysis, we accounted for the additional investment required up to 2018 when it is expected that the program will have achieved the necessary scale and become sustainable. Over the period 2007-2014, HCHC was involved in the operational activities of three insurance programs in Nigeria (one rural – the KSHI, and two urban), we allocated HCHC’s overheads over the three programs according to volume of enrolees during this period. In 2014, the urban programs were terminated and from that moment onwards, HCHC’s overheads had been allocated to the KSHI program exclusively.

The second component refers to expenses upgrading facilities entering the KSHI program. All clinics in the program have to meet a certain minimum criteria of quality of care. Therefore, in most cases clinics require upgrading before entering the program.

The last component refers to technical assistance delivered by PharmAccess Foundation. Technical assistance consists of: health plan design, data-management, participation in the SafeCare program and additional quality improvement trainings. In particular, all clinics in KSHI programs are enrolled in the SafeCare program, in which they are assessed and receive quality improvement reports. In this analysis, expenses related to operational research and impact evaluation were not taken into account.

We accounted for start-up costs of the program in 2006 (enrolment began in 2007). A contract with Kwara State Government has been signed to take over the responsibility of the program at the end of 2018. Therefore, the above-service costs have been calculated over the 2006-2018 period. For the years 2006 to 2013, financial records audited by accountants and consistent with subsidy statements from donors were used. The amounts related to 2014 to 2016 are based on budgets. From 2016 to 2018, the figures are based on projections. Although the Kwara State Governor signed a contract to enrol 600.000 people in the program by 2018, a lower target has been assumed in this analysis (a conservative assumption). As of December 2014, the program had reached 40% of its enrolment target. Therefore, as a conservative assumption, it is assumed that 40% of the original target will be reached by 2018 (40%*600,000 = 243,000 people enrolled in December 2018), which represents the basis for our projections.

Table S10. Overview of the above-service program costs for the period 2006-2018, divided by realised costs vs projected costs (US$ 2012).

|  | **2006-2013**  **(realised costs)** | **2014-2018**  **(projected costs)** | **2006-2018** |
| --- | --- | --- | --- |
| Operational costs | 10.91 | 9.98 | 10.24 |
| Upgrading costs | 1.05 | 0.76 | 0.84 |
| Technical assistance costs | 22.63 | 9.38 | 13.02 |
| **Total** | **34.60** | **20.13** | **24.10** |

The weighted average (weighted by number of enrolees per year) of above-service program costs per enrolee per year, over the full program period, was estimated at US$2012 24.10. We added this as a mark-up to all patients in the intervention scenario only.

## Reference scenario

### Overview of costs

For the main analysis, ANC, uncomplicated and complicated delivery (in EOC) costs were assumed to be the same as those in the KSHI scenario: ANC, US$2012 12.4-61.5; uncomplicated delivery, US$2012 9.65-27.2; and complicated delivery, US$2012 46.7-53.3. These were in accordance to costs for ANC (US$2005 23.75-25.10; US$2012 44.8-47.3), and a conservative estimate compared to the costs found in the literature for normal delivery at a facility offering EOC (US$2005 18.74-18.82; US$2012 35.3-35.5), and complicated deliveries (from US$2005 23.7-150.8; US$2012 44.6-284.3) at comprehensive obstetric care facility (29). This particular assumption was assessed by varying the costs of base case services.

The treatment of fistula has been estimated to cost from US$2012 190.9 to 382.8 (42). These costs include direct costs related to consumables and staff time for treatment and postoperative care. We also included a mark-up of 20-30% as suggested in the literature for Nigeria (43,44) to account for indirect costs such as managerial, administrative and support staff, utilities and communication costs.

The cost for treatment of severe anaemia was estimated following published guidelines (32) using an ingredients costing approach. The costs included treatment for six months post-partum, three outpatient visits, and two tests. Cost for drugs and tests were calculated from the data collected in the KSHI scenario and the outpatient visit cost was sourced from WHO-CHOICE (45). We assumed 74.8% of women access postnatal care in Kwara State (5) in the standard of care scenario while the proportion of women accessing postnatal care in the KSHI scenario was set at the same as those accessing ANC.

# Sensitivity analysis

To assess the sensitivity of our results to the performance of the base case, we defined three alternative base case scenarios. Assumptions made are presented in table S10.

Table S11 - Alternative base case scenarios: assumptions.

| **Scenario 1** | | |
| --- | --- | --- |
| **access to ANC in SoC** | Base case | 0.6-0.7 |
|  | Base case, scenario 1 | 0.8-0.9 |
| **delivery in health facility in SoC** | Base case | 0.4-0.6 |
|  | Base case, scenario 1 | 0.65-0.7 |
| **Scenario 2** | | |
| **cost ANC in SoC** | Base case | 12.4-61.5 |
|  | Base case, scenario 2 | 44.8-47.3 |
| **cost delivery no complications in SoC** | Base case | 9.7-27.2 |
|  | Base case, scenario 2 | 35.3-35.5 |
| **cost delivery complications in SoC** | Base case | 46.7-53.3 |
|  | Base case, scenario 2 | 44.6-284.3 |
| **Access to EOC if delivery in HF** | Base case | 0.9-0.95 |
|  | Base case, scenario 2 | 1 |
| **rr of hypertensive disorders if ANC** | Base case | 0.123 |
|  | Base case, scenario 2 | 0.41 |
| **Scenario 3** | | |
| **access to ANC in SoC** | Base case | 0.6-0.7 |
|  | Base case, scenario 3 | 0.8-0.9 |
| **delivery in health facility in SoC** | Base case | 0.4-0.6 |
|  | Base case, scenario 3 | 0.65-0.7 |
| **cost ANC in SoC** | Base case | 12.4-61.5 |
|  | Base case, scenario 3 | 44.8-47.3 |
| **cost delivery no complications in SoC** | Base case | 9.7-27.2 |
|  | Base case, scenario 3 | 35.3-35.5 |
| **cost delivery complications in SoC** | Base case | 46.7-53.3 |
|  | Base case, scenario 3 | 44.6-284.3 |
| **Access to EOC if delivery in HF** | Base case | 0.9-0.95 |
|  | Base case, scenario 3 | 1 |
| **rr of hypertensive disorders if ANC** | Base case | 0.123 |
|  | Base case, scenario 3 | 0.41 |

ANC, antenatal care; SoC, standard of care; EOC, emergency obstetric care; HF, health facility; rr, risk reduction.

We performed a series of one-way sensitivity analyses, all assumptions and detailed results are presented in table S11, additional results (these correspond to figure 2: one-way sensitivity analysis in the main text).


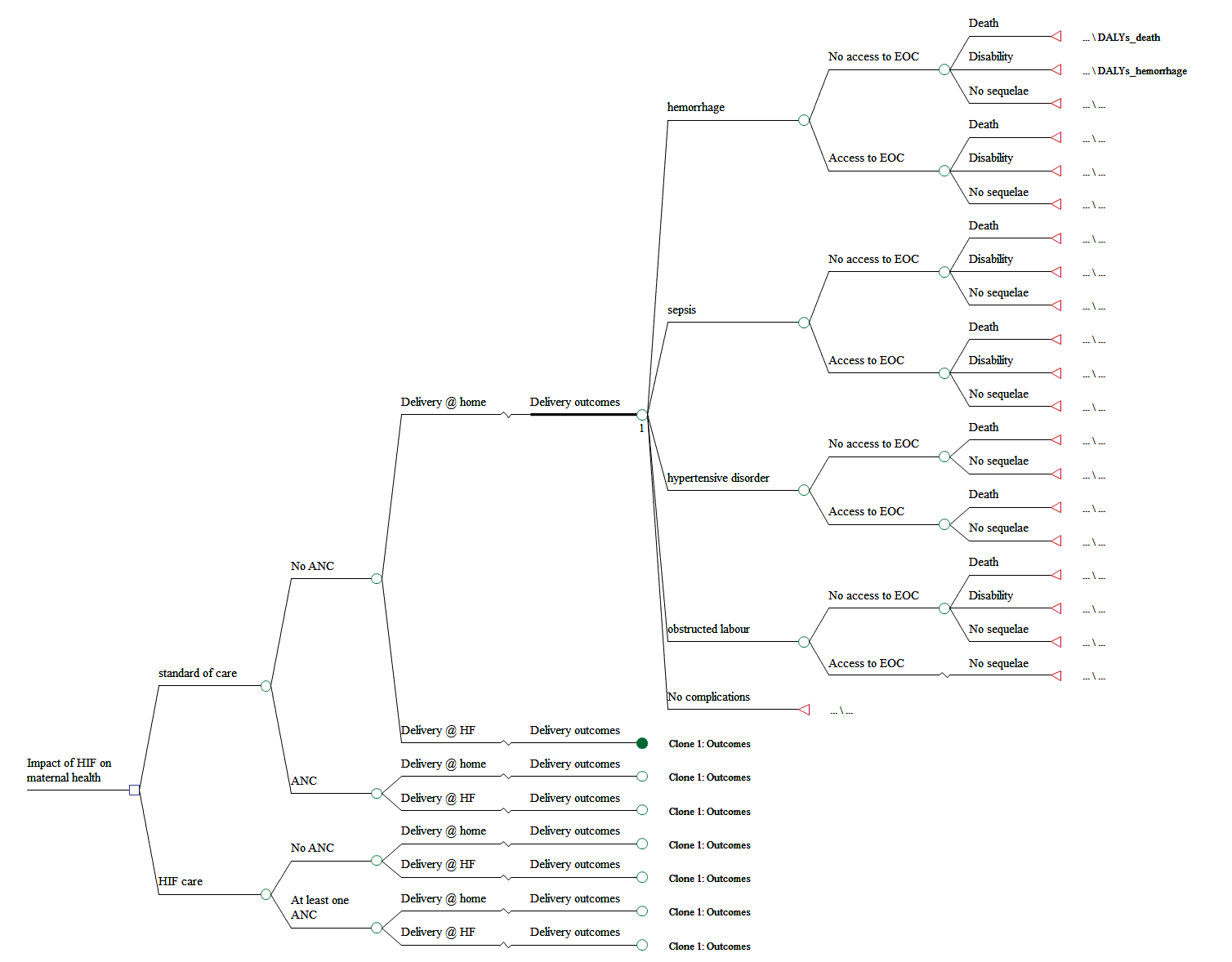
Figure S1 – schematic of model pathways.

Figure S2: Cost-effectiveness acceptability curves.


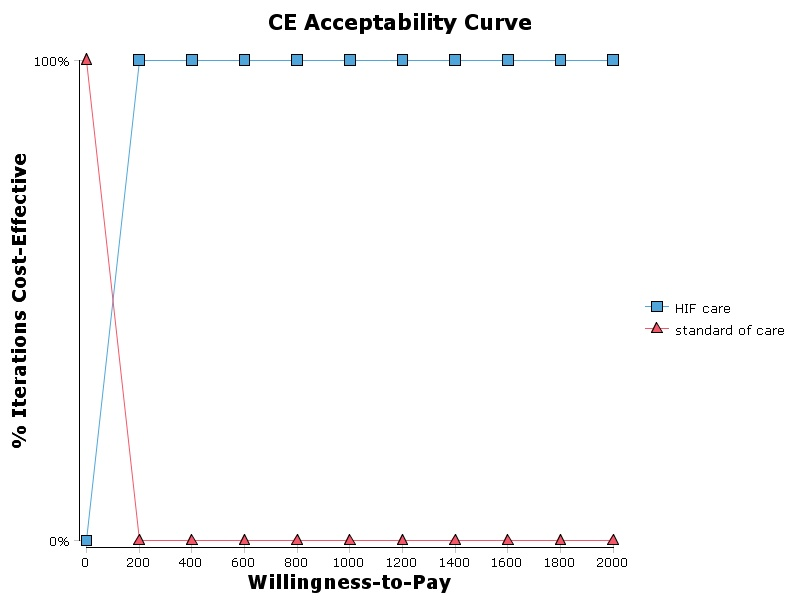


# Additional results

Table S12 – One-way sensitivity analysis, all.

| **Parameter** |  | **Assumption** | **ICER, mean** |
| --- | --- | --- | --- |
| **Discount rate** | Base case | 0.03 | 46.36 |
|  | high | 0.1 | 110.38 |
|  | low | 0 | 26.85 |
| **p haemorrhage** | Base case | 0.051 | 46.36 |
|  | high | 0.2 | 43.57 |
|  | low | 0.001 | 47.22 |
| **p sepsis** | Base case | 0.017 | 46.36 |
|  | high | 0.1 | 35.06 |
|  | low | 0.001 | 49.95 |
| **p hypertensive disorders** | Base case | 0.085 | 46.36 |
|  | high | 0.2 | 20.61 |
|  | low | 0.001 | 374.84 |
| **p obstructed labour** | Base case | 0.06 | 46.36 |
|  | high | 0.2 | 46.73 |
|  | low | 0.001 | 46.07 |
| **p death, haemorrhage** | Base case | 0.028 | 46.36 |
|  | high | 1 | 38.6 |
|  | low | 0 | 47.86 |
| **p death, sepsis** | Base case | 0.73 | 46.36 |
|  | high | 1 | 37.68 |
|  | low | 0 | 257.4 |
| **p death, hypertensive disorders** | Base case | 0.083 | 46.36 |
|  | high | 1 | 24.21 |
|  | low | 0 | 50.51 |
| **p death, obstructed labour** | Base case | 0.007 | 46.36 |
|  | high | 1 | 32.84 |
|  | low | 0 | 46.39 |
| **p anaemia** | Base case | 0.12 | 46.36 |
|  | high | 0.5 | 46.93 |
|  | low | 0.001 | 45.69 |
| **p infertility** | Base case | 0.1 | 46.36 |
|  | high | 0.5 | 66.68 |
|  | low | 0.001 | 39.09 |
| **p fistula** | Base case | 0.14 | 46.36 |
|  | high | 0.5 | 46.24 |
|  | low | 0.001 | 46.49 |
| **cost fistula repair** | Base case | 190.953 | 46.36 |
|  | high | 572.859 | 46.29 |
|  | low | 63.651 | 46.41 |
| **cost anaemia treatment** | Base case | 9.806628097 | 46.36 |
|  | high | 29.41988429 | 46.36 |
|  | low | 3.268876032 | 46.36 |
| **above service cost** | Base case | 24.1 | 46.36 |
|  | high | 250 | 338.81 |
|  | low | 5 | 21.63 |
| **duration disability, anaemia** | Base case | 0.5y | 46.36 |
|  | high | 2y | 46.31 |
|  | low | 1mo | 46.37 |
| **duration disability, fistula** | Base case | 36.6y | 46.36 |
|  | high | 50y | 46.35 |
|  | low | 1y | 46.41 |
| **duration disability, infertility** | Base case | 21.4y | 46.36 |
|  | high | 40y | 46.33 |
|  | low | 1y | 46.4 |

P, probability.

Figure S2: Acceptability curve.


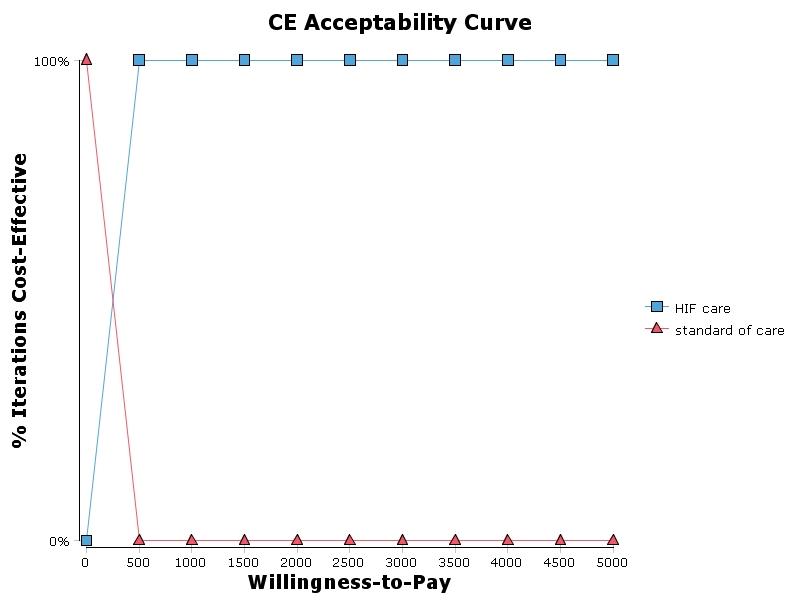


# References

1. Gustafsson-Wright E, Schellekens O. Achieving universal health coverage in Nigeria one state at a time: a public-private partnership community-based health insurance model. Washington DC: Brookings; 2013.

2. Carroli G, Rooney C, Villar J. How effective is antenatal care in preventing maternal mortality and serious morbidity? An overview of the evidence. Paediatr Perinat Epidemiol. 2001 Jan;15 Suppl 1:1–42.

3. World Health Organization, Reproductive Health and Research. Pregnancy, childbirth, postpartum, and newborn care: a guide for essential practice. Geneva: World Health Organization; 2003.

4. Federal Ministry of Health [Nigeria]. Standard Treatment Guidelines. Abuja, Nigeria: Federal Ministry of Health in collaboration with WHO, EC, DFID; 2008.

5. National Population Commission (NPC) [Nigeria], ICF International. Nigeria Demographic and Health Survey 2013. Abuja, Nigeria, and Rockville, Maryland, USA: NPC and ICF International; 2014.

6. Adewemimo AW, Msuya SE, Olaniyan CT, Adegoke AA. Utilisation of skilled birth attendance in Northern Nigeria: A cross-sectional survey. Midwifery. 2014 Jan;30(1):e7–e13.

7. Doctor HV, Findley SE, Cometto G, Afenyadu GY. Awareness of critical danger signs of pregnancy and delivery, preparations for delivery, and utilization of skilled birth attendants in Nigeria. J Health Care Poor Underserved. 2013 Feb;24(1):152–70.

8. Federal Ministry of Health [Nigeria]. National HIV & AIDS and Reproductive Health Survey, 2012 (NARHS Plus). Federal Ministry of Health Abuja, Nigeria; 2013.

9. Carroli G, Villar J, Piaggio G, Khan-Neelofur D, Gülmezoglu M, Mugford M, et al. WHO systematic review of randomised controlled trials of routine antenatal care. Lancet. 2001 May 19;357(9268):1565–70.

10. Brals D, Wit FWNM. The impact of health insurance and medical facility-upgrades on institutional delivery among women in rural Nigeria: A cohort study. Unpublished.

11. World Health Organization, United Nations Population Fund, United Nations Children’s Fund. Guidelines for monitoring the availability and use of obstetric services. New York: UNICEF : WHO : UNFPA; 1997.

12. Erim DO, Kolapo UM, Resch SC. A Rapid Assessment of the Availability and Use of Obstetric Care in Nigerian Healthcare Facilities. PLoS ONE. 2012 Jun 22;7(6):e39555.

13. Saidu R, August EM, Alio AP, Salihu HM, Saka MJ, Jimoh AAG. An assessment of essential maternal health services in Kwara State, Nigeria. Afr J Reprod Health. 2013 Mar;17(1):41–8.

14. Murray CJL, Ezzati M, Flaxman AD, Lim S, Lozano R, Michaud C, et al. GBD 2010: design, definitions, and metrics. Lancet. 2012 Dec 15;380(9859):2063–6.

15. World Bank, Disease Control Priorities Project. Disease control priorities in developing countries. 2nd ed. Jamison DT, editor. New York : Washington, DC: Oxford University Press ; World Bank; 2006. 1401 p.

16. Carroli G, Cuesta C, Abalos E, Gulmezoglu AM. Epidemiology of postpartum haemorrhage: a systematic review. Best Pract Res Clin Obstet Gynaecol. 2008 Dec;22(6):999–1012.

17. Calvert C, Thomas SL, Ronsmans C, Wagner KS, Adler AJ, Filippi V. Identifying regional variation in the prevalence of postpartum haemorrhage: a systematic review and meta-analysis. PLoS ONE. 2012;7(7):e41114.

18. AbouZahr C. Global burden of maternal death and disability. Br Med Bull. 2003;67:1–11.

19. Begley CM, Gyte GML, Devane D, McGuire W, Weeks A. Active versus expectant management for women in the third stage of labour. Cochrane Database Syst Rev. 2011;(11):CD007412.

20. Dolea C, Abou Zahr C. Global burden of obstructed labour in the year 2000 [Internet]. World Health Organization. Available from: http://www.who.int/healthinfo/statistics/bod_obstructedlabour.pdf

21. Abalos E, Cuesta C, Grosso AL, Chou D, Say L. Global and regional estimates of preeclampsia and eclampsia: a systematic review. Eur J Obstet Gynecol Reprod Biol. 2013 Sep;170(1):1–7.

22. Duley L, Gülmezoglu AM, Henderson-Smart DJ, Chou D. Magnesium sulphate and other anticonvulsants for women with pre-eclampsia. Cochrane Database Syst Rev. 2010;(11):CD000025.

23. McDonald SD, Lutsiv O, Dzaja N, Duley L. A systematic review of maternal and infant outcomes following magnesium sulfate for pre-eclampsia/eclampsia in real-world use. Int J Gynaecol Obstet. 2012 Aug;118(2):90–6.

24. Ekele BA. Use of magnesium sulfate to manage pre-eclampsia and eclampsia in Nigeria: overcoming the odds. Ann Afr Med. 2009 Jun;8(2):73–5.

25. Maternal Health and Safe Motherhood Programme. Mother-Baby Package: Implementing Safe Motherhood in Countries. Geneva: World Health Organization; 2006.

26. Seale AC, Mwaniki M, Newton CRJC, Berkley JA. Maternal and early onset neonatal bacterial sepsis: burden and strategies for prevention in sub-Saharan Africa. Lancet Infect Dis. 2009 Jul;9(7):428–38.

27. Dolea C, Stein C. Global burden of maternal sepsis in the year 2000 [Internet]. World Health Organization. Available from: http://www.who.int/healthinfo/statistics/bod_maternalsepsis.pdf

28. Kaye DK, Kakaire O, Osinde MO. Systematic review of the magnitude and case fatality ratio for severe maternal morbidity in sub-Saharan Africa between 1995 and 2010. BMC Pregnancy Childbirth. 2011;11:65.

29. Erim DO, Resch SC, Goldie SJ. Assessing health and economic outcomes of interventions to reduce pregnancy-related mortality in Nigeria. BMC Public Health. 2012;12:786.

30. Guerrier G, Oluyide B, Keramarou M, Grais R. High maternal and neonatal mortality rates in northern Nigeria: an 8-month observational study. Int J Womens Health. 2013;5:495–9.

31. Fox-Rushby JA, Hanson K. Calculating and presenting disability adjusted life years (DALYs) in cost-effectiveness analysis. Health Policy Plan. 2001 Sep;16(3):326–31.

32. Stoltzfus RJ, Dreyfuss ML. Guidelines for the use of iron supplements to prevent and treat iron deficiency anemia. Washington, DC: ILSI Pr.; 1999.

33. National Population Commission (NPC) [Nigeria], ICF Macro. Nigeria Demographic and Health Survey 2008. Abuja, Nigeria: National Population Commission and ICF Macro; 2009.

34. World Health Organization. Global Health Observatory Data Repository: Life expectancy: Life tables Nigeria [Internet]. Available from: http://apps.who.int/gho/data/view.main.61200

35. Umoiyoho A, Inyang-Etoh E, Abah G, Abasiattai A, Akaiso O. Quality of life following successful repair of vesicovaginal fistula in Nigeria. Rural and Remote Health. 2011;11:1734.

36. Kwara State Government [Internet]. Available from: http://www.kwarastate.gov.ng/index.php

37. Federal Republic of Nigeria. Population and Housing Census 2006. Population distribution by sex, state, LGA & senatorial district [Internet]. Available from: http://www.ibenaija.org/uploads/1/0/1/2/10128027/vol_03_table_dsx_lgapop_by_sdistrict-pdf.pdf

38. Hendriks M, Brewster L, Wit F, Bolarinwa O, Odusola A, Redekop W, et al. Cardiovascular disease prevention in rural Nigeria in the context of a community based health insurance scheme: QUality Improvement Cardiovascular care Kwara-I (QUICK-I). BMC Public Health. 2011;11(1):186.

39. World Health Organization, UNICEF, Aga Khan Foundation. Cost analysis in primary health care: a training manual for programme managers. Geneva: World Health Organization; 1994. 147 p.

40. Economic evaluation. Maidenhead ; New York: Open University Press; 2005. 253 p.

41. Conteh L, Walker D. Cost and unit cost calculations using step-down accounting. Health Policy Plan. 2004 Mar;19(2):127–35.

42. Fistula Care. Estimating Costs to Provide Fistula Services in Nigeria and Ethiopia: Key Findings. New York: EngenderHealth/Fistula Care; 2012.

43. Johns B, Sigurbjörnsdóttir K, Fogstad H, Zupan J, Mathai M, Tan-Torres Edejer T. Estimated global resources needed to attain universal coverage of maternal and newborn health services. Bull World Health Organ. 2007 Apr;85(4):256–63.

44. Johns B, Baltussen R, Hutubessy R. Programme costs in the economic evaluation of health interventions. Cost Eff Resour Alloc. 2003 Feb 26;1(1):1.

45. WHO-CHOICE. WHO-CHOICE unit cost estimates for service delivery [Internet]. CHOosing Interventions that are Cost Effective (WHO-CHOICE). [cited 2014 Aug 1]. Available from: http://www.who.int/choice/country/country_specific/en/
